# Prevalence of hyperuricemia and the relationship between serum uric acid and obesity: A study on Bangladeshi adults

**DOI:** 10.1371/journal.pone.0206850

**Published:** 2018-11-01

**Authors:** Nurshad Ali, Rasheda Perveen, Shahnaz Rahman, Shakil Mahmood, Sadaqur Rahman, Shiful Islam, Tangigul Haque, Abu Hasan Sumon, Rahanuma Raihanu Kathak, Noyan Hossain Molla, Farjana Islam, Nayan Chandra Mohanto, Shaikh Mirja Nurunnabi, Shamim Ahmed, Mustafizur Rahman

**Affiliations:** 1 Department of Biochemistry and Molecular Biology, Shahjalal University of Science and Technology, Sylhet, Bangladesh; 2 Department of Biochemistry and Molecular Biology, Gonoshasthaya Samaj Vittik Medical College, Gono University, Savar, Dhaka, Bangladesh; 3 Department of Biochemistry and Molecular Biology, Bangabandhu Sheikh Mujibur Rahman Science and Technology University, Gopalganj, Bangladesh; University of Colorado Denver School of Medicine, UNITED STATES

## Abstract

**Background and objectives:**

Recent studies have shown that hyperuricemia is commonly associated with dyslipidemia, cardiovascular diseases, hypertension and metabolic syndrome. Elevated serum uric acid has been demonstrated to be associated with obesity in the adult population in many countries; however, there is still a lack of evidence for the Bangladeshi population. The aims of this study were to evaluate the prevalence of hyperuricemia and determine the relationship between serum uric acid (SUA) and obesity among the Bangladeshi adults.

**Methods:**

In this cross-sectional study, blood samples were collected from 260 adults (142 males and 118 females) and analyzed for SUA and lipid profile. All participants were categorized as underweight (n = 11), normal (n = 66), overweight (n = 120) and obese (n = 63) according to the body mass index (BMI) scale for the Asian population. Based on SUA concentration the participants were stratified into four quartiles (Q1: < 232 μmol/L, Q2: 232–291 μmol/L, Q3: 292–345 μmol/L and Q4: > 345 μmol/L).

**Results:**

The mean age and BMI of the participants were 32.5 ± 13.3 years and 24.9 ± 3.8 kg/m^2^, respectively. The average level of SUA was 294 ± 90 μmol/L with a significant difference between males and females (p < 0.001). Overall, the estimated prevalence of hyperuricemia was 9.3% with 8.4% in male and 10.2% in female participants. There were significant increases in the prevalence of obesity (17.4%, 22.2%, 28.6% and 31.8%, respectively, p < 0.01 for trend) across the SUA quartiles. A multiple logistic regression analysis revealed that SUA quartiles were independently associated with the presence of obesity (p < 0.01).

**Conclusion:**

Present study indicates a significant positive relationship between SUA and obesity among the Bangladeshi adults. Therefore, routine measurement of SUA is recommended in obese individuals to prevent hyperuricemia and its related complications.

## Introduction

Serum uric acid (SUA) is the end-product of purine metabolism in humans [1]. Approximately two-thirds of SUA are produced endogenously and the remaining as a result of diet abundant purines [2,3]. Uric acid is excreted primarily via the kidneys (> 70%) with a smaller portion via intestinal and biliary secretion [2]. Abnormalities in SUA metabolism and its decreased excretion by the kidneys are one of the major causes of hyperuricemia and gout development [4,5]. An increased exogenous consumption of proteins and endogenous production of uric acid in obese persons are additional factors that lead to hyperuricemia [6].

In the last few decades, the prevalence of hyperuricemia is increasing rapidly in the world population. Emerging evidence shows that hyperuricemia is prevalent not only in the developed countries [7,8] but also increasing in the low and middle-income countries with a high frequency [9,10]. Lifestyle factors like obesity, purine abundant diet and alcohol intake are determined to be independent predictors for the development of hyperuricemia [11,12,13]. According to epidemiological studies on metabolic syndrome, SUA was found to be positively related to several indices, such as BMI, waist circumference, and dyslipidemia [14,15]. Thus, hyperuricemia is considered to be a common lifestyle disorder related to obesity in humans [7,14]. Several epidemiological studies indicated that hyperuricemia is associated with a number of diseases including diabetes mellitus, dyslipidemia, obesity, hypertension, cardiovascular diseases, and metabolic syndrome [16,17,18,19]. Among these disorders, obesity is one of the global health issues increasing rapidly in the world community. Obesity not only causes severe effects to individual health but also imposes a significant burden on the healthcare system [20]. Moreover, it has been recognized as an associated risk factor with a variety of adverse health consequences including diabetes, hypertension and elevated SUA [7]. Hyperuricemia and its relation with obesity have been documented in previous studies. A significant positive association has been found between SUA and obesity in the adult population of China [21,22,23,24], Japan [25], India [7,26], Pakistan [27] and Iraq [28]. However, there is a lack of studies on the association between obesity and SUA for the Bangladeshi population. The aims of this study were to evaluate the prevalence of hyperuricemia and assess the relationship between SUA and obesity among the Bangladeshi adults.

## Materials and methods

### Participants and study design

This study was a cross-sectional design and conducted between August and October 2017. The study consisted of 260 participants (142 males and 118 females) recruited from academic and non-academic staffs and students of Gonoshasthaya Samaj Vittik Medical College, Gono University, located in Dhaka district and Shahjalal University of Science and Technology in Sylhet district of Bangladesh. All participants were informed about the study and gave their written consent before inclusion in the study. This study was approved by the ethics committee of Gonoshasthaya Samaj Vittik Medical College, Gono University, Dhaka 1344, Bangladesh. Pregnant women, lactating mothers and the individuals with having a history of surgical operation, drug addiction, anti-hypertensive and anti-hyperuricemic drugs intake, history of hepatic, renal or severe cardiac diseases, and gout have been excluded from the study.

### Anthropometric measurements

Basis anthropometric measurements were recorded by trained health technicians in a structured questionnaire form. Anthropometric data such as individual height, weight, waist circumference (WC) and hip circumference (HC) and other lifestyle information were obtained using the standard procedure described elsewhere [29]. Height was measured to the nearest 0.1 cm and weight was measured to the nearest 0.1 kg by modern electronic digital LCD weighing machines (Beurer 700, Germany) wearing light clothing and no shoes. The scales were calibrated every day against a standard (20 kg). The body mass index (BMI) was calculated as the weight (in kg) divided by the body height (in m^2^). Waist circumference was measured midway between the lowest border of the ribs and iliac crest in the horizontal plane and hip circumference was measured at a level parallel to the floor, at the largest circumference of the buttocks to the nearest 0.5 cm with anthropometric tape. The waist-to-hip ratio (WHR) was then calculated by dividing waist circumference by hip circumference. The quality of anthropometric data was confirmed by repeated measurements in presence of investigators.

### Laboratory measurements

Fasting blood samples (5 mL) were drawn from the participants under strict aseptic precautions and allow them to clot and centrifuged at 3000 rpm for 15 minutes for serum separation. Serum uric acid (SUA), triglycerides (TG), total cholesterol (TC), high-density lipoprotein (HDL) and low-density lipoprotein (LDL) were measured separately by colorimetric methods according to the manufacturer's protocols (Human Diagnostic, Germany) with a biochemistry analyzer (Humalyzer 3000, USA). All procedures were done by trained staff and accuracy of the analysis was maintained through standard calibration on regular basis. All serum samples were analyzed in duplicate and the mean value was used in the calculation.

### Diagnostic criteria

Based solely on SUA levels, there is no universally-accepted definition for hyperuricemia. In present study, hyperuricemia was defined if participants having their SUA concentration was > 7.0 mg/dL (416.4 μmol/L) in men or > 6.0 mg/dL (356.9 μmol/L) in women [30,31]. These cutoff values were selected as they are generally used in clinical laboratories and have been proposed in previous studies in relation to metabolic syndrome and cardiovascular disease outcomes to define hyperuricemia [30]. Serum uric acid levels were categorized into four quartiles to compare the prevalence of obesity and its association with SUA quartiles. Based on diagnostic criteria for obesity for Asian populations recommended by the WHO, we categorized BMI into four groups: underweight (< 18.5 kg/m^2^), normal weight (18.5–23.0 kg/m^2^), overweight (23.0–27.5 kg/m^2^), and obese (≥ 27.5 kg/m^2^) [32]. Abdominal or central obesity was defined as a WC ≥ 90 cm for men and ≥ 80 cm for women and WHR ≥ 0.90 and ≥ 0.80 for male and female, respectively [33].

### Statistical analysis

All data were analyzed by IBM SPSS statistics version 23. Independent sample t-test (two-tailed) was done to assess the differences between male and female cohort for anthropometric and baseline variables. Interrelationships between anthropometric, baseline variables and SUA were assessed by Pearson’s correlation coefficient test. One-way ANOVA was performed to determine differences among the groups. The binary logistic regression was applied to assess the association between SUA quartiles and obesity. The values in tables were presented as mean ± standard deviation otherwise noted. A level of alpha 0.05 was assigned for statistical significance.

## Results

### Baseline characteristics of the study cohorts

The basic characteristics of the study cohorts are summarized in Table 1. Of the 260 subjects, 54.6% were males, and 45.4% were females. The mean age of the participants was 32.5 ± 13.3 years (range 18–80 years), with a significant difference between males and females (p < 0.01).

**Table 1:** Baseline characteristics and SUA level of the study cohort.

	Total	Male	Female	P-value
Number (n)	260	142 (54.6%)	118 (45.4%)	
Age (years)	32.5 ± 13.3 (80)	34.8 ± 15.3 (80)	29.7 ± 9.6 (62)	0.006
Height (cm)	160.0 ± 7.9 (177)	165.5 ± 5.3 (177)	153.4 ± 4.7 (165)	0.000
Weight (kg)	64.0 ± 10.9 (92)	67.7 ± 9.3 (88)	59.3 ± 11.0 (92)	0.000
WC (cm)	84.6 ± 8.7 (116)	85.7 ± 7.8 (106)	82.9 ± 9.8 (116)	0.047
HC (cm)	93.8 ± 7.7 (124)	92.9 ± 5.6 (107)	95.1 ± 10.1 (124)	0.081
BMI (kg/m^2^)	24.9 ± 3.8 (37)	24.6 ± 3.4 (34)	25.2 ± 4.3 (37)	0.319
SUA (μmol/L)	294.0 ± 90.0 (826)	321.7 ± 95.4 (826)	260.3 ± 69.7 (440)	0.000
TG (mg/dl)	154.0 ± 90.6 (673)	172.3 ± 90.5 (360)	133.1 ± 86.5 (673)	0.003
TC (mg/dl)	138.6 ± 49.0 (257)	132.4 ± 54.6 (257)	145.5 ± 41.0 (253)	0.067
HDL (mg/dl)	43.6 ± 12.5 (82)	40.5 ± 9.8 (64)	47.9 ± 14.6 (82)	0.000
LDL (mg/dl)	75.4 ± 39.4 (210)	71.2 ± 41.1 (210)	81.4 ± 36.2 (189)	0.109

Data are presented as mean ± SD. Values in parentheses indicate maximum level of the parameter. P value obtained from independent sample t-test in comparison the gender group.

The average BMI for all subjects was 24.9 ± 3.8 kg/m^2^ with no significant difference between gender groups (p > 0.05). Mean value of WC was 84.6 ± 8.7 with a significant difference between male and female (p < 0.05) subjects. A significant difference also observed for the mean level of serum SUA (p < 0.001), TG (p < 0.01) and HDL (p < 0.001) in the gender group.

### Prevalence of hyperuricemia among the participants

Based on the diagnostic criteria, 24 participants were identified as hyperuricemic individuals. Overall, the estimated prevalence of hyperuricemia was 9.3% with 8.4% in male and 10.2% in female participants (Table 2). In the BMI categories, the prevalence of hyperuricemia was 1.9% in normal, 1.6% in overweight and 5.8 in the obesity group. The mean level of SUA was 275 ± 61 μmol/L (max 416 μmol/L) and 460 ± 86 μmol/L (max 826 μmol/L) in the non-hyperuricemic and hyperuricemic group, respectively.

**Table 2:** Comparison of baseline characteristics between non-hyperuricemic and hyperuricemic subjects.

	Non-hyperuricemia (n = 236)	Hyperuricemia (n = 24)	p-value
Male (n = 142)	130 (91.6%)	12 (8.4%)	-
SUA (μmol/L)	303 ± 65 (416)	529 ±140 (826)	0.000
Female (n = 118)	106 (89.8%)	12 (10.2%)	-
SUA (μmol/L)	246 ± 57 (357)	390.7± 30.8 (440)	0.000
Age (years)	32.5 ± 13.1	32.8 ± 15.3	0.910
WC (cm)	84.0 ± 8.6	90.7 ± 7.9	0.006
HC(cm)	93.3 ± 7.5	98.8 ± 8.9	0.011
BMI (kg/m^2^)	24.5 ± 3.8	26.9 ± 4.5	0.005
TG (mg/dl)	150.5 ± 92.2	162.6 ± 67.0	0.047
TC (mg/dl)	136.8 ± 49.6	162.1 ± 34.0	0.042
HDL (mg/dl)	47.3 ± 12.6	42.6 ± 10.9	0.045
LDL (mg/dl)	74.6 ± 39.2	88.1 ± 42.0	0.320

Values are presented as mean ± SD. SUA level indicated in parentheses as the maximum. Hyperurecmia was defined as the SUA level in men ≥416.4 (7mg/dl) and in women ≥356.9 (6mg/dl) by Sui et al. [30].

A significant difference was observed for BMI (p < 0.01), WC (p < 0.01) and HC (p < 0.05) between non-hyperuricemic and hyperuricemic group (Table 2).

### Correlation of SUA with BMI, WC and HC

**Fig 1:**
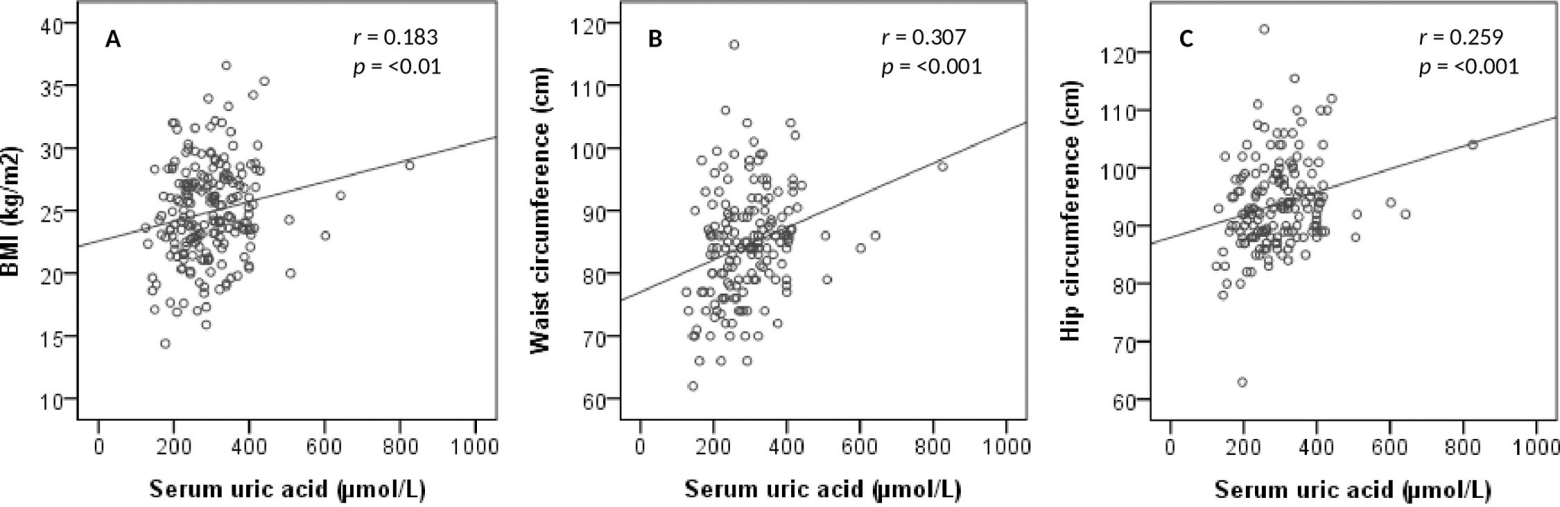
Correlation between serum uric acid with BMI (A), waist circumference (B) and hip circumference (C).

**Fig 2:**
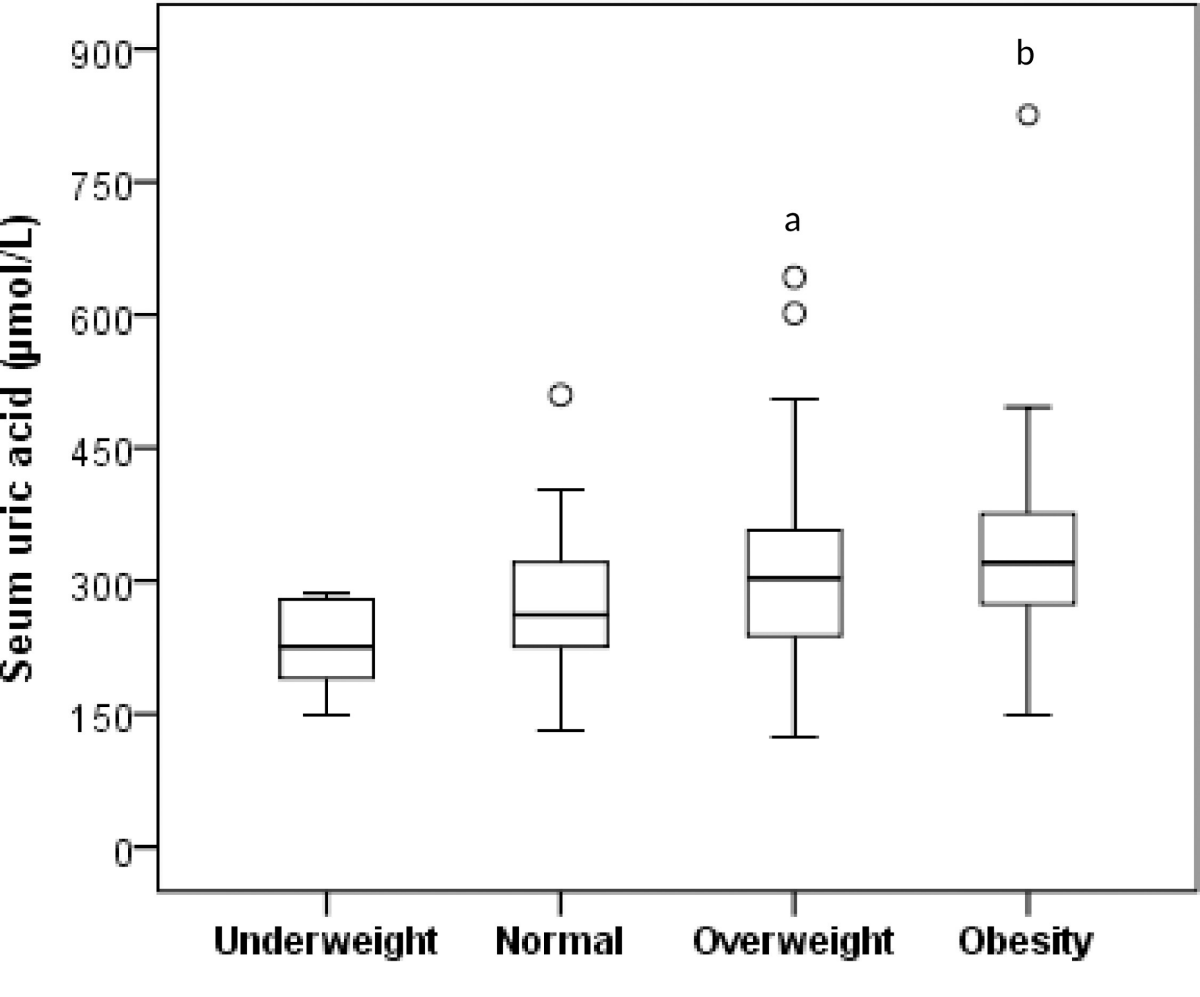
Level of serum uric acid in different BMI (kg/m^2^) groups. Based on diagnostic criteria for obesity for Asian populations recommended by the WHO, BMI has been categorized into four groups: underweight (<18.5), normal weight (18.5–23.0), overweight (23.0–27.5), and obese (≥27.5) [32]. ^a^p<0.05 and ^b^p<0.01 when compared to the underweight category.

Fig 1 presents the correlation of SUA with BMI, WC and HC for all subjects. After adjusting for age and sex, the correlation analysis demonstrated the strong positive correlation of SUA with BMI (p < 0.01), WC (p < 0.001) and HC (p < 0.001). There were some participants with the highest SUA levels not having the highest BMI (Fig 1). We predict that this might happen under some conditions such as intake of high purine containing food and beverages or breakdown of the cell which increases excessive uric acid level in blood. Uric acid may also increase in blood when it doesn’t filter out enough through the kidneys. The correlation level between SUA and BMI did not change after removing such individuals from the analysis. When BMI was categorized into underweight, normal, overweight and obesity groups, a significant difference for SUA levels were found in overweight (p < 0.05) and obesity (p < 0.01) group when compared to underweight group (Fig 2).

### Serum uric acid quartiles and comparison of obesity between the quartiles

The baseline characteristic of the participants according to SUA quartiles are shown in Table 3. The subjects with higher SUA quartiles were more likely to be men as expected. After adjustment of age and sex, the BMI, WC, HC, SUA, TG, TC and LDL levels were progressively increased and HDL level was progressively decreased across the SUA quartiles (Table 3).

**Table 3:** Characteristics of the subjects according to SUA (μmol/L) quartiles.

	Q1 < 232	Q2 232–291	Q3 292–345	Q4 > 345	F	p-value
Number (n)	66	67	65	62	-	-
Gender (m/f)	22/44	31/36	41/24	48/14	-	-
Age (years)	35.1 ± 14.8	30.6 ± 12.9	32.8 ± 12.4	31.2 ± 12.7	1.21	0.307
WC (cm)	81.5 ± 9.7	83.0 ± 9.6	87.0 ± 7.1	87.5 ± 6.5	5.26	0.002
HC (cm)	90.6 ± 7.4	93.7 ± 8.7	95.5 ± 6.9	96.4 ± 6.8	4.83	0.003
BMI (kg/m^2^)	23.9 ± 3.9	24.2 ± 3.9	25.6 ± 3.7	25.9 ± 3.5	3.27	0.003
SUA (μmol/L)	198 ± 29	263 ± 18	329 ± 16	413 ± 35	2.03	0.000
TG (mg/dl)	135.2 ± 83.3	131.2 ± 71.3	175.3 ± 109.2	182.7 ± 85.1	4.20	0.007
TC (mg/dl)	126.7 ± 46.3	128.9 ± 48.2	145.7 ± 49.1	155.3 ± 48.5	3.82	0.038
HDL (mg/dl)	44.8 ± 13.4	44.5 ± 14.1	43.3 ± 11.0	39.6 ± 10.2	2.31	0.046
LDL (mg/dl)	68.2 ± 36.0	67.5 ± 42.5	82.3 ± 34.7	87.6 ± 42.4	1.47	0.053

Values are presented as mean ± SD. P-values are obtained from one-way ANOVA.

**Fig 3:**
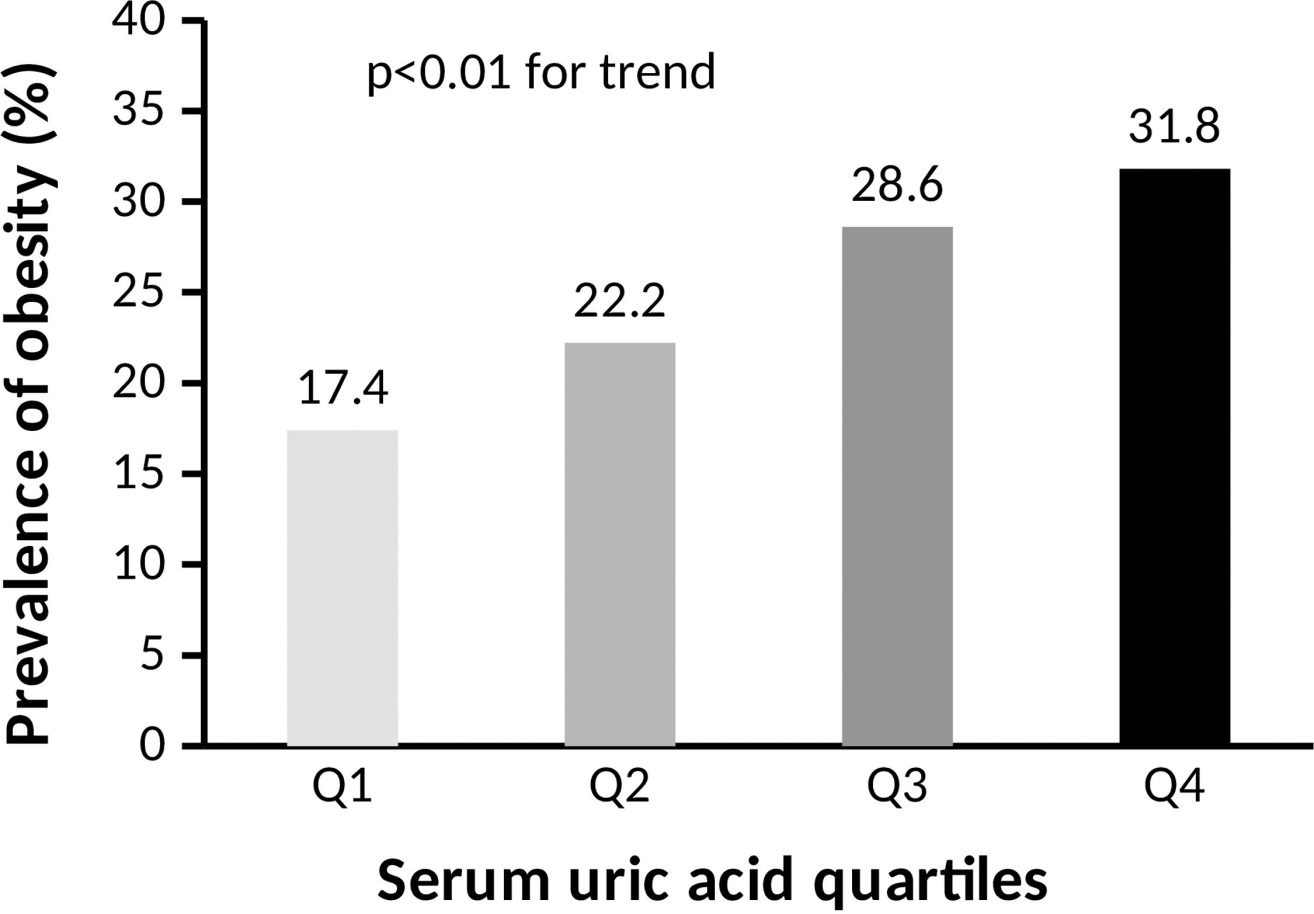
Comparison of obesity between the SUA quartile groups.

The prevalence of obesity was significantly increased with the increasing SUA quartile (17.4%, 22.2%, 28.6% and 31.8% for the first, second, third and fourth quartiles, respectively, p < 0.01 for trend) (Table 4 and Fig 3).

**Table 4:** Prevalence of underweight, normal, overweight and obesity between the SUA quartiles.

		Prevalence n, (%)				Total	p-values for trend
		Underweight	Normal	Overweight	Obesity		
	Number (n)	11	66	120	63	260	
Male	Q1	4 (36.3)	6 (9.1)	9 (7.5)	4 (6.3)	23 (8.8)	< 0.01
	Q2	2 (18.2)	12 (18.2)	13 (10.8)	5 (7.9)	32 (12.3)	
	Q3	0 (0.0)	6 (9.1)	25 (20.8)	8 (12.7)	39 (15.0)	
	Q4	0 (0.0)	5 (7.6)	32 (26.7)	11 (17.5)	48 (18.5)	
Female	Q1	3 (27.3)	17 (25.7)	18 (15.0)	7 (11.1)	45 (17.3)	< 0.01
	Q2	2 (18.2)	10 (25.7)	14 (11.7)	9 (14.3)	35 (13.5)	
	Q3	0 (0.0)	6 (9.1)	6 (5.0)	10 (15.9)	22 (8.5)	
	Q4	0 (0.0)	4 (6.1)	3 (2.5)	9 (14.3)	16 (6.1)	

The p-value is for trend when the prevalence has compared between the SUA quartiles.

### Association of SUA quartiles with obesity

The association of SUA quartiles with obesity for all participants is presented in Table 5. After adjusting for potential confounder’s like age and sex (Model 1), the SUA quartiles were independently associated with an increased prevalence of obesity (p < 0.01 for trend). After additional adjustments for age, sex, TG and TC (Model 2) and for age, sex, TG, TC, HDL and LDL (Model 3), the SUA quartiles were still independently and significantly associated with the increased prevalence of obesity (p < 0.01 for Model 2 and 3). Compared with the lowest SUA quartile, the OR for obesity in the highest SUA quartile group was 1.82 (95% CI, 1.39–2.44) after adjusting multiple confounding variables (Model 3).

**Table 5:** Association of serum uric acid quartiles with obesity.

	OR (95% CI)				P values for trend
	Q1	Q2	Q3	Q4	
Model 1	1	1.43 (1.15–1.78)	2.07 (1.66–2.58)	3.32 (2.64–4.20)	< 0.01
Model 2	1	1.36 (1.10–1.70)	1.84 (1.50–2.33)	2.92 (2.32–3.72)	< 0.01
Model 3	1	1.20 (0.91–1.58)	1.46 (1.15–1.96)	1.82 (1.39–2.44)	< 0.01

The binary logistic regression was done to access the association between SUA quartiles and obesity. Model 1: age, sex and BMI were selected. Model 2: age, sex, TG and TC were selected. Model 3: age, sex, TG, TC, HDL and LDL were selected.

## Discussion

Obesity in the last decade become a global problem and has been recognized as a risk factor with a variety of clinical conditions and adverse health consequences; hyperuricemia is one of these conditions [28]. Nowadays, obesity and hyperuricemia and its complications, such as metabolic syndrome and cardiovascular diseases, have raised serious concern for public health in the international community because of their high prevalence, health consequences and substantial economic burden [21]. Several epidemiological studies have assessed the relationship between SUA and obesity in different population; however, to our knowledge, this is the first study that focused on SUA and obesity for the Bangladeshi population. In present study, we explored the prevalence of hyperuricemia and the potential association of SUA with obesity.

In our study, SUA levels were higher in males than females, and similar results were found in previous studies [23,34]. In this investigation, the incidence of hyperuricemia was 9.3% (8.4% in men and 10.2% in women) (Table 2), which was in accordance with the worldwide prevalence rate reported to be ranging from 2.6% to 36% in different populations [35]. In Asia, the prevalence of hyperuricemia in mainland China was 13.3% (19.4% in men and 7.9% in women) and 25.8% (34.5% in men and 11.6% in women) in Japan [36]. As expected, the prevalence of hyperuricemia found in our study is close to that in most developing countries; for example, it is 10.6% in Thailand (18.4% in men and 7.8% in women) [37], 12.1% in Turkey (19.0% in men and 5.8% in women) [38], and 8.4% among Saudi men and women [39].

Results of the present study confirmed the strong association of SUA with obesity and central obesity in the Bangladeshi adults after adjustment of age, sex, BMI and lipid profile. This association was stronger in female participants in both overweight and obesity group, which was consistent with the findings of previous studies [23,40,41]. We observed that the prevalence of obesity steadily increased across the SUA quartiles, and the SUA levels tightly and independently related to obesity even after controlling other risk factors such as age, sex and lipid profile (Table 4 and Fig 3). A positive association was also found between increased SUA and overweight, waist circumference. Consistent with our findings, several epidemiological studies have also shown a positive association of SUA with overweight and obesity in different population. For example, in a 10-year follow-up study, BMI was found to be significantly increased with increasing SUA levels in all race-sex-groups [42]. Furthermore, not only elevated SUA levels were associated with the increased risk of obesity, but obesity was also related with higher risk of hyperuricemia. For example, Tanaka et al. [14] found that BMI was significantly correlated with SUA levels in Japanese adult twins, after adjusting genetic and environmental factors in both genders. Another study by Wang et al. [15] reported a positive relationship between BMI and SUA levels among healthy individuals in Jiangsu province of China. In more recent studies, a significant positive relationship was observed between SUA levels and obesity in population of China [21,22,23,24], Japan [25], India [7,26], Pakistan [27] Iraq [28] and United States [43]. Although a positive association between obesity and SUA levels has been reported in previous studies; the mechanism by which how uric acid is increased in obesity has not well elucidated yet. Obesity may be linked to SUA levels involving two factors: overproduction and poor renal excretion. A study conducted among the participants with visceral fat obesity indicates that increased levels of uric acid are strongly influenced by its overproduction with a decrease in urinary urate excretion and clearance [44]. Moreover, visceral fat accumulation induces an elevated influx of plasma free fatty acids into live and hepatic portal vein which stimulates the synthesis of triglycerides followed by an associated surge in uric acid production through the activation of uric acid synthesis pathway [14,45].

There were some limitations to our study. First, the cross-sectional nature of the data cannot prove cause-effect relationships between SUA and obesity. Second, the sample size in this study was relatively small, therefore our findings do not represent for the entire population of Bangladesh. Moreover, the findings of our study may not apply to other ethnic populations. It has been observed that SUA is a significant determinant of changes in BMI, and SUA levels may predict the subsequent weight gain [46]. However, the underlying mechanism by which SUA is increased in obese individuals still remains to explore. More studies need to be done in order to establish the mechanism of the association between SUA and obesity in humans.

## Conclusion

Results of this study indicate a significant positive association between SUA and obesity among the adult population in Bangladesh. Therefore, routine measurement of SUA is recommended in obese individuals to prevent hyperuricemia and its related complications.
